# Peppered and rare – Gastric and Duodenal Pseudomelanosis: A case series

**DOI:** 10.12669/pjms.333.12995

**Published:** 2017

**Authors:** Sami Samiullah, Hadi Bhurgri, Arooj Babar, Fatima Samad, Moaz M. Choudhary, Michael Demyen

**Affiliations:** 1 Sami Samiullah, Department of Gastroenterology and Hepatology, University of Missouri, Columbia, MO, USA; 2 Hadi Bhurgri, Department of Gastroenterology and Hepatology, Rutgers University, Newark, NJ, USA; 3 Arooj Babar, Department of Medicine, Rutgers University, Newark, NJ, USA; 4 Fatima Samad, Department of Medicine, St Luke’s Medical Center, Milwaukee, WI, USA. Rutgers University, Newark, NJ, USA; 5 Moaz M. Choudhary, Department of Medicine, Rutgers University, Newark, NJ, USA; 6 Michael Demyen, Department of Gastroenterology and Hepatology, Rutgers University, Newark, NJ, USA

**Keywords:** Duodenum, Melanosis, Pseudomelanosis, Stomach

## Abstract

Upper Gastrointestinal (GI) pseudomelanosis is an uncommon entity characterized by endoscopic visualization of speckled dark mucosal pigmentation. While described in the rectum and colon, ‘melanosis’ or more aptly ‘pseudomelanosis’ is rare in the duodenum and exceedingly rare in the stomach. Five cases of pseudomelanosis were encountered at our department. Four females and one male were diagnosed, with a mean age of 70 years. All patients exhibited duodenal pseudomelanosis, with one demonstrating gastric antral pseudomelanosis as well. Common features among these patients included iron deficiency anemia, hypertension, chronic kidney disease, hydralazine use and iron supplementation. Biopsy specimens stained at least partially positive for iron and stains for calcium and copper were negative. Histochemical analysis revealed the pigment of pseudomelanosis to be mainly iron sulfide, exhibiting unpredictable staining patterns, hypothesized to be secondary to varying sulfur content and iron oxidation. It is visualized as dark deposits in macrophages at the tips of the duodenal villi. Upper GI pseudomelanosis remains a poorly understood finding, weakly associated with chronic kidney disease, diabetes, hypertension, iron supplements and anti-hypertensive medications. While the pathogenesis, clinical and prognostic significance remains unclear, it is thus far considered a benign condition.

## INTRODUCTION

Pseudomelanosis of the upper gastrointestinal (GI) tract is an uncommon entity characterized by endoscopic visualization of speckled dark pigmentation of the mucosa.^1^ It should be noted that while ‘melanosis’ has been described previously in the gastrointestinal tract in colon and rectum, duodenal pseudomelanosis is rare with gastric pseudomelanosis being exceedingly rare with only few reported cases.^2^,^3^ We present four cases of endoscopic and histological pseudomelanosis duodeni with one case representing both pseudomelanosis of duodenum and the gastric antrum.

## CASE PRESENTATION

We encountered five cases at our endoscopy center (Table-I). The chart review was approved by the Institutional Review Board at Rutgers University. The medical history, demographics and histopathology were reviewed. The demographics, clinical features and laboratory values are summarized in Table-I.

**Table-I:** Demographics, clinical features and laboratory values of patients with pseudomelanosis.

*Case*	*Age/Sex*	*Location*	*Indication for EGD*	*Hemoglobin (g/dL)*	*Creatinine clearance (ml/min)*	*Ejection Fraction*	*Medical Co-morbidities*
1	89/M	Antrum/ Duodenum	GERD	7.9	31.3	55 %	Iron deficiency anemia, essential hypertension & chronic kidney disease
2	59/F	Duodenum	Dysphagia	8.9	63	55%	Iron deficiency anemia and essential hypertension.
3	64/F	Duodenum	GI Bleed	8.4	15.2	60%	Iron deficiency anemia, essential hypertension & chronic kidney disease
4	64/F	Duodenum	Dysphagia	9.2	42.6	75 %	Iron deficiency anemia, essential hypertension & chronic kidney disease
5	74/F	Duodenum	GI Bleed	9.0	42.3	35 %	Iron deficiency anemia, essential hypertension & chronic kidney disease

**Fig.1:**
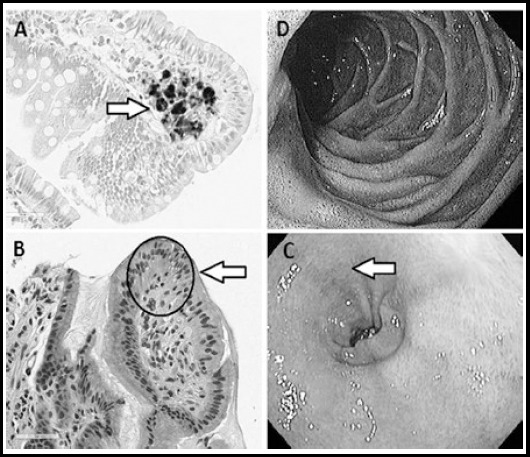
a) High power (40x) duodenum with positive Perl’s iron staining in the lamina propria. b) High power (40x) stomach Hematoxylin and Eosin showing a dark pigment deposit. c) Endoscopic view of duodenum. d) Endoscopic view of antrum.

The mean patient age was 70 years. Four were female and one was male. All had endoscopic findings of pseudomelanosis in the duodenum with one case having pseudomelanosis extending into the antrum of stomach (Table-I). Four patient had iron deficiency anemia, essential hypertension and chronic kidney disease while one patient had only essential hypertension and iron deficiency anemia. Two patients had diabetes, four had dyslipidemia, two reported dysphagia and three had COPD. All patients were taking hydralazine and oral ferrous sulfate. Three patients were on Lasix and three were taking inhaled bronchodilators and inhaled steroids for Chronic Obstructive Pulmonary Disease (COPD). Biopsies were taken in all cases, and all were negative for Helicobacter Pylori *(H. Pylori)*. The histopathologic features are summarized in Table-II. The endoscopic pigmentation and the histologic slides, characterizing the pigment deposits are depicted in Fig.1.

**Table-II:** Histopathologic features.

*Case*	*Location*	*Iron Perl’s Stain*	*Copper Rhodanine Stain*	*Calcium – Von Kossa Stain*
1	Antrum/Duodenum	Positive	Negative	Negative
2	Duodenum	Positive	Negative	Negative
3	Duodenum	Partial Positive	Negative	Negative
4	Duodenum	Partial Positive	Negative	Negative

## DISCUSSION

Pseudomelanosis of the duodenum, first described in 1976, refers to the endoscopic visualization of speckled dark pigmentation of the mucosa.^1^ Histologic analysis reveals a granular pigment in the macrophage lysosomes in the lamina propria around the tips of the duodenal villi. Initially described as melanosis duodeni, it was more aptly changed to pseudomelanosis in later years as more became known about the pigment itself.^2^,^3^

It should be noted that while ‘melanosis’ is described elsewhere in the gastrointestinal tract, including the colon and rectum, duodenal pseudomelanosis is rare with gastric pseudomelanosis being exceedingly rare. Also, the nature of the pigment is different, with colonic pigments being more commonly composed of melanin and lipofuscin, with iron deposits being less common. Melanin does not contain iron, and furthermore melanocytes are not present in the gastrointestinal mucosa. In contrast, the heterogeneous pigment found in duodenal pseudomelanosisis thought to be represent compounds like hemosiderin, Lipomelanin and li-pofuscin, with greater iron content.^2^

Histochemical stains and electron probe analysis reveal the pigment to be composed mainly of iron sulfide, although varying amounts of sulfur, calcium, potassium, aluminum, magnesium and silver are also seen.^3^,^4^ It must also be noted that it is more commonly seen histologically, with some cases showing no gross visualization on endoscopy.^2^ Staining patterns have been known to be unpredictable, with certain cases showing different staining patterns on repeat endoscopies. This is thought to result from varying forms of oxidation of the ferrous compounds and incorporation of sulfur. In our cases (Table-II), Perl’s stain was either positive or partially positive, and stains for calcium and copper were negative. Although since the stains for calcium and copper are dark, the deposits being dark themselves might confound the staining results.

Epidemiologically, it is described mostly in females in the 6^th^ to 7^th^ decade of life with co-morbidities such as hypertension, diabetes, chronic kidney disease and end stage renal disease and gastric hemorrhage, although cases in adolescents have also been described.^5^,^6^ While it is described frequently in patients with end stage renal disease, it is not exclusively seen with chronic kidney disease, with some cases having normal renal function.^3^,^7^ Certain medications such as iron supplements, hydralazine, hydrochlorothiazide, propranolol and furosemide have been associated with the condition.^8^,^9^ Although oral iron therapy seems to have the strongest association, pseudomelanosis has been described in its absence. One case also noted pseudomelanosis duodeni in a 48 year old female with none of the medications or medical conditions described above, who presented solely with dyspepsia. We also reviewed charts for the presence of heart failure, and none of the patients had congestive heart failure.

When reviewing all the reported cases, it is noted that the most common indications for esophagogastroduodenoscopy (EGD) in order are, GI bleeding, gastro-esophageal reflux disease (GERD), dyspepsia and dysphagia. Two of our patients underwent EGD to evaluate dyspepsia with no structural cause being identified. Gastric pseudomelanosis has only been described a handful of times in literature.^10^-^13^ All these cases show speckled dark pigmentation in the gastric antrum. It is unclear whether this is an extension of duodenal pseudomelanosis or a different pathophysiology. While the pathogenesis is not clearly understood, no clinical or prognostic significance has been described and no surveillance or follow up guidelines are identified to date.

## CONCLUSION

Pseudomelanosis of the upper GI tract remains a rare finding loosely associated with chronic kidney disease, diabetes and hypertension, with certain medications including iron supplements and anti hypertensives, and yet none of these features appear to be essential in its development. The pathogenesis remains unclear and no clinical or prognostic significance has thus been determined. Of the cases described in the literature, no long term follow up has been reported.

### Author’s Contribution

***SS:*** Data acquisition, collection, manuscript writing and revision of final draft.

***HB, AB and FS:*** Data collection, manuscript writing and revision of final draft.

***MC:*** Data collection, manuscript writing and revision of final draft. MD: Manuscript writing and revision of final draft.

***SS*** takes the responsibility and is accountable for all aspects of the work in ensuring that questions related to the accuracy or integrity of any part of the work are appropriately investigated and resolved.
